# Binding Analysis of Sf-SR-C MAM Domain and Sf-FGFR Ectodomain to Vip3Aa

**DOI:** 10.3390/insects15060428

**Published:** 2024-06-06

**Authors:** Chenghai Wang, Min Li, Xiling Chen, Shilong Fan, Jun Lan

**Affiliations:** 1 School of Biomedical Sciences, Hunan University, Changsha 410082, China; wangch0529@hnu.edu.cn; 2 Beijing Advanced Innovation Center for Structural Biology, School of Life Sciences, Tsinghua University, Beijing 100084, China; helen2019@mail.tsinghua.edu.cn (M.L.); xlchen9057@126.com (X.C.); fanshilong@mail.tsinghua.edu.cn (S.F.)

**Keywords:** Vip3Aa, Sf-SR-C, Sf-FGFR, crystal structure, SPR, structure docking

## Abstract

**Simple Summary:**

Because the Scavenger Receptor-C (SR-C) and Fibroblast Growth Factor Receptor (FGFR) of *Spodoptera frugiperda* (Sf-SR-C and Sf-FGFR) have formerly been determined as the receptors of the *Bacillus thuringiensis* Vip3Aa toxin in Sf9 cells, the mechanism of Vip3Aa binding to its receptor urgently needs to be demonstrated. In this study, we solved the crystal structure of the Sf-MAM domain at a resolution of 2.25 Å. Through structural docking analysis, we found that the Sf-MAM domain may not interact with the Vip3Aa protein. Using surface plasmon resonance (SPR) binding analysis, we further verified that both the Sf-MAM domain and the Sf-FGFR ectodomain could not bind to the Vip3Aa protein in vitro. Thus, the invasion mechanism of the Vip3Aa toxin into its host cells needs further study.

**Abstract:**

*Bacillus thuringiensis* Vip3Aa has been widely used in transgenic crops to resist the erosion of insects. The Scavenger Receptor-C (SR-C) and Fibroblast Growth Factor Receptor (FGFR) of *Spodoptera frugiperda* (Sf-SR-C and Sf-FGFR) have formerly been identified as the cell receptors of Vip3Aa. However, the interaction mechanism of Vip3Aa binding to Sf-SR-C or Sf-FGFR is still unknown. Here, we purified the MAM domain of Sf-SR-C (Sf-MAM) and the Sf-FGFR ectodomain expressed extracellularly by Sf9 cells. We then solved the crystal structure of the Sf-MAM domain. Structure docking analysis of the Sf-MAM and Vip3Aa C-terminal domain (CTD) excluded the possibility of the two proteins binding. A further surface plasmon resonance (SPR) assay also revealed that the Sf-MAM and Sf-FGFR ectodomain could not bind to the Vip3Aa protein. Our results have raised the urgency of determining the authentic cell receptor for Vip3Aa.

## 1. Introduction

Vegetative insecticidal proteins (Vips) are secreted by *Bacillus thuringiensis* (Bt) during the vegetative growth stage [1,2]. In the new classification of the Vip family, the Vip family protein can be divided into three groups based on its structural homology, namely Vpb, Vpa and Vip [3]. The proteins in Vpb1 (formerly known as Vip1) and Vpa (formerly known as Vip2) usually function as a binary toxin to exert insecticidal activity against Coleoptera and Hemiptera pests [4]. The Vip (formerly known as Vip3) group contains over 110 proteins and is most widely studied. The proteins in the Vip group mainly bind to the receptors on midgut epithelial cells to induce pore formation and apoptosis, and eventually the death of Lepidoptera pests (such as *Spodoptera frugiperda*) [5,6]. Vip proteins have a wide insecticidal spectrum and high insecticidal activity, and thus are considered as a second-generation pesticide [7]. As Vip proteins share no sequence homology and insecticidal process with the known insecticidal crystal proteins (ICPs), the stack strategy of Vip and ICP co-expression has been wildly used in the development of commercial transgenic crops, including maize and cotton [8,9].

The structures of Vip3Aa and Vip3Bc1 were previously solved by cryo-electron microscopy (cryo-EM), with Protein Data Bank (PDB) codes of 6TFJ and 6YRF, respectively. These two similar structures indicated that Vip proteins exist as a tetramer protoxin composed of five domains [10,11]. In Vip3Aa, the five domains were Domain 1 (Ala12-Thr190), Domain 2 (Glu208-Ile322), Domain 3 (Thr329-Thr518), Domain 4 (Asn537-Ile667) and Domain 5 (Ile679-Lys789). This research showed that Domain 1 was essential to the stability of the protoxin tetramer, while Domain 2 and Domain 3 were involved in cell receptor binding [12,13,14]. However, studies regarding the mechanism implicated in the insecticidal function of Vip3Aa, especially the receptor binding mechanism, are not sufficient.

In 2018, researchers in China found that the scavenger receptor-C (SR-C) of *S. frugiperda* (Sf-SR-C) is the cell receptor for Vip3Aa [15]. They showed that Sf-SR-C could mediate the toxicity of Vip3Aa through promoting the internalization of Vip3Aa into *S. frugiperda* cells (Sf9), and that the complement control protein (CCP) domain and MAM domain of Sf-SR-C (Sf-CCP and Sf-MAM) were directly involved in the interaction with Vip3Aa. Using a microscale thermophoresis (MST) assay, the binding affinity of Sf-CCP and Sf-MAM to Vip3Aa was estimated as 2.19 ± 1.55 μM and 463 ± 117 nM, respectively [15]. Later, they found that the fibroblast growth factor receptor (FGFR) of *S. frugiperda* (Sf-FGFR) may serve as a novel receptor for Vip3Aa [16]. Sf-FGFR could co-localize with Vip3Aa on the surface of Sf9 cells, and the binding affinity of the Sf-FGFR ectodomain to Vip3Aa was 1.43 ± 0.87 μM, detected also by an MST assay [16]. However, a recent study established the Sf-SR-C and Sf-FGFR knock-out Sf9 strains using the CRISPR/Cas9 system. The results showed that the sensitivity of the Sf9 normal strain to Vip3Aa toxin was not significantly changed compared with the two knock-out strains. Therefore, they concluded that the Sf-SR-C and Sf-FGFR genes were not essential for Vip3Aa to exert insecticidal toxicity on Sf9 cells [17].

Regarding this controversy, determining the interaction of Vip3Aa with Sf-SR-C or Sf-FGFR at the molecular level in vitro becomes very necessary. Here, we report an expression strategy used to purify a large-scale soluble fraction of the Sf-MAM domain and Sf-FGFR ectodomain, which is suitable for the binding analysis expressed extracellularly by Sf9 cells. We then successfully solve the crystal structure of the Sf-MAM domain, dock this structure to the Vip3Aa C-terminal domain (CTD) structure and exclude the possibility of interaction between the two proteins. The surface plasmon resonance (SPR) assay further demonstrated that the MAM domain of Sf-SR-C and the ectodomain of Sf-FGFR could not bind to the Vip3Aa protein. These results will pave the way for the future determination of the Vip3Aa cell receptor and the demonstration of the insecticidal mechanism of Vip3Aa.

## 2. Materials and Methods

### 2.1. Protein Expression and Purification

The full-length Vip3Aa protein (Met1-Lys789) was cloned into the pET15b vector with an N-terminal 6×His tag. The protein was expressed in BL21 (DE3), which was induced by 0.2 mM of IPTG at 18 °C after the OD_600_ reached 1.0. The supernatant was collected after ultrasonic crushing and loaded onto a Hitrap Ni-chelating column equilibrated with buffer A (20 mM of HEPES, 500 mM of NaCl, pH 7.2). After washing with 50 volumes of buffer A containing 20 mM of imidazole, the protein was eluted with buffer A containing 400 mM of imidazole. Then, the protein was loaded onto a Superdex 200 10/300 gel filtration column pre-equilibrated with buffer A. The fractions were collected for the binding assay.

The Sf-MAM domain (residues Thr139-Leu320) and the Sf-FGFR ectodomain (residues Gln27-Ser403) were expressed using the Bac-to-Bac system (Invitrogen, Carlsbad, CA, USA) in Sf9 cells. Briefly, the genes encoding the two proteins were cloned onto a pFastBac-dual vector with a GP67 signal peptide at the N-terminal and a C-terminal 6×His tag. The vector was transformed into DH10bac competent cells to obtain the recombined bacmid. Then, the bacmid was transfected into Sf9 cells. The high-titer baculovirus was obtained seven days later to further transfect the Sf9 cells for protein expression. Three days after transfection, the supernatant was collected and buffer-exchanged into buffer A and then loaded onto a Hitrap Ni-chelating column equilibrated with buffer A. After washing with 50 volumes of buffer A containing 20 mM of imidazole, the protein was eluted with buffer A containing 400 mM of imidazole. The elution was then concentrated and loaded onto a Superdex 200 10/300 gel filtration column pre-equilibrated with buffer A. The fractions containing the Sf-MAM domain or Sf-FGFR ectodomain were collected for the binding assay. For crystallization, the Sf-MAM domain protein was concentrated to 15 mg/mL. The purity of the proteins was judged by SDS-PAGE.

### 2.2. Crystallization of the Sf-MAM Domain

Crystal screening was carried out by the sitting-drop vapor diffusion method using Hampton Research crystallization screen kits and 96-well Intelli plates. Typically, 400 nL sitting drops were obtained by mixing 200 nL of the Sf-MAM protein and 200 nL of the reservoir solution. The reservoir wells contained 60 μL of solution. The crystals of the Sf-MAM domain appeared about two weeks later at 291 K from the No. 5 condition of the PEG/Ion screen kit (Hampton Research, Aliso Viejo, CA, USA), which contained 0.2 M magnesium chloride, 20% polyethylene glycol 3350 and pH 5.9.

### 2.3. Data Collection and Structure Determination

The crystals of the Sf-MAM domain were mounted on a cryo-loop and flash-cooled with liquid nitrogen in a cryoprotection solution consisting of 0.2 M magnesium chloride, 20% polyethylene glycol 3350, pH 5.9 and 20% glycerol. The diffraction data were collected at the SSRF-BL17U1 beamline at 100 K. The diffraction data were processed using HKL3000 [18]. The structure of the Sf-MAM domain was determined by molecular replacement using the structure of Tyrosine Phosphatase Mu (PDB code: 2C9A) as a search model. The structure was further refined by COOT and PHENIX [19,20]. The values of Ramachandran favored, Ramachandran allowed and Ramachandran outliers were 94.62%, 5.38% and 0.00%, respectively. The structure was uploaded to PDB with a PDB code of 8YT7. All the structure figures were generated by Pymol [21].

### 2.4. Structure Docking of Sf-MAM Domain to the Vip3Aa CTD

ZDOCK SERVER was used to dock the structure of the Sf-MAM domain to the Vip3Aa CTD structure [22]. After uploading the two structures, ten putative binding models (complexes 1–10) were then generated, with estimated ZDOCK scores ranging from 1199.9 to 1322.7. The high ZDOCK score indicated the high level of credibility of the binding models.

### 2.5. Surface Plasmon Resonance Spectroscopy

The Sf-MAM domain or Sf-FGFR ectodomain was immobilized on a channel of a CM5 chip using Biacore 8K^+^ (GE Healthcare, Chicago, IL, USA), with a response unit (RU) of nearly 1000. Serial dilutions (3.125–3200 nM) of Vip3Aa were flowed through, with a running buffer containing 20 mM of HEPES, 500 mM of NaCl, pH 7.2, and 0.05% (*v*/*v*) Tween 20. The association and dissociation time were set as 90 s and 180 s, respectively. The final raw curves were fitted to a 1:1 binding model using the Kinetics mode in Biacore Evaluation Software v5.0.18.22102 (GE Healthcare, Chicago, IL, USA).

## 3. Results

### 3.1. Protein Purification

**Figure 1:**
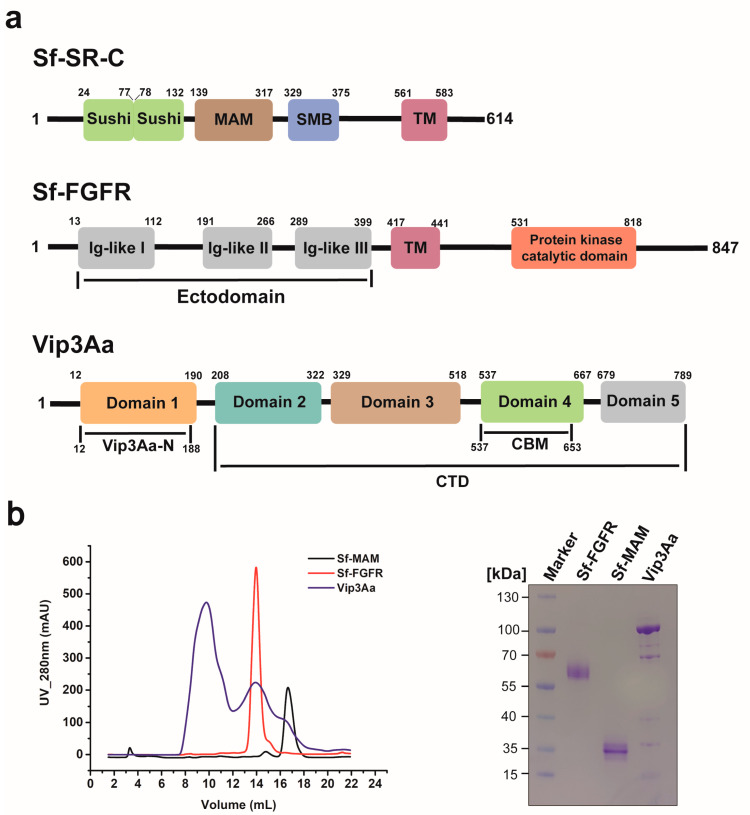
Protein purification of Vip3Aa, Sf−MAM and Sf−FGFR. (**a**) Schematic presentation of Sf-SRC, Sf-FGFR and Vip3Aa protein. (**b**) Protein purification of Vip3Aa, Sf-MAM domain and Sf-FGFR ectodomain. The size exclusion curves for Vip3Aa, Sf-FGFR ectodomain and Sf-MAM domain are colored in cyan, red and black, respectively.

The Sf-SR-C and Sf-FGFR are single transmembrane proteins in Sf9 cells. A recent study demonstrated that the ectodomain of Sf-FGFR or the extracellular MAM domain of Sf-SR-C are the main domains that interact with Vip3Aa [15,16]. Therefore, we chose to use the Bac-to-Bac system to express the Sf-MAM domain or the Sf-FGFR ectodomain (Figure 1a and Figure S1), not the entire protein, in our study. The two proteins were secreted in the supernatant mediated by an N-terminal GP67 signal peptide. The molecular weights of the Sf-MAM domain and Sf-FGFR ectodomain were estimated as 21.0 kDa and 43.3 kDa, respectively. The Sf-MAM domain and the Sf-FGFR ectodomain were expressed as a monomer, indicated by size exclusion chromatography and SDS-PAGE (Figure 1b). The increase in the molecular weights of the Sf-MAM domain and Sf-FGFR ectodomain on SDS-PAGE was due to glycosylation (the Sf-MAM domain contained three glycosylation sites and the Sf-FGFR ectodomain contained ten glycosylation sites). The Vip3Aa protein contained five domains (Domains 1–5) and the full-length protein was expressed in the *Escherichia coli* system and purified separately (Figure 1). In order to understand the interaction of Vip3Aa with the two receptors, the in vitro binding test of Vip3Aa with the Sf-MAM domain or the Sf-FGFR ectodomain expressed by Sf9 cells may be more convincing.

### 3.2. The Structure of Sf MAM Domain

**Figure 2:**
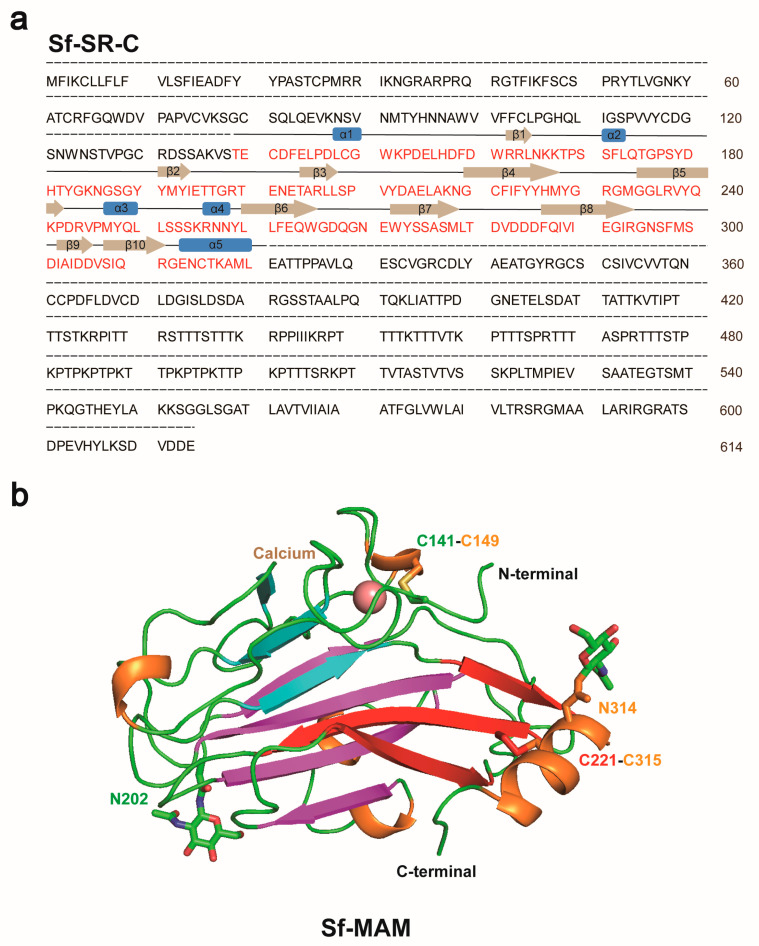
Structure of the Sf−MAM domain. (**a**) Amino acid sequence of the Sf-SR-C protein. The sequence of the Sf-MAM domain is marked in red. The secondary structures are indicated above. (**b**) Crystal structure of the Sf-MAM domain. The three pairs of β-strands are colored in cyan, magenta and red, respectively. Short α-helixes are colored in orange. Cysteine and asparagine residues are shown as sticks. Calcium ion is shown as a salmon sphere.

Using X-ray crystallography, we determined the structure of the Sf-MAM domain at a resolution of 2.25 Å. The space group of the structure was P3_2_21. The *R*_work_ and *R*_free_ factors of the structure were 26.47% and 26.97%, respectively (Table S1). Only one molecule was present in one asymmetric unit. The overall structure contained ten β-strands, which could be divided into three pairs and five short α-helixes (Figure 2 and Figure S2). Although the expression construct of the Sf-MAM domain contained three glycosylation sites (Asn186, Asn202 and Asn314), only two glycan chains that linked to the Asn202 and Asn314 residues were built in the final structure (Figure 2b and Figure S2). Two disulfide bonds were formed by Cys141-Cys149 and Cys221-Cys315 near both termini of the protein. A calcium atom was present near the N-terminal of the structure (Figure 2b and Figure S2).

### 3.3. Structure Docking of the Sf-MAM Domain to the Vip3Aa CTD

**Figure 3:**
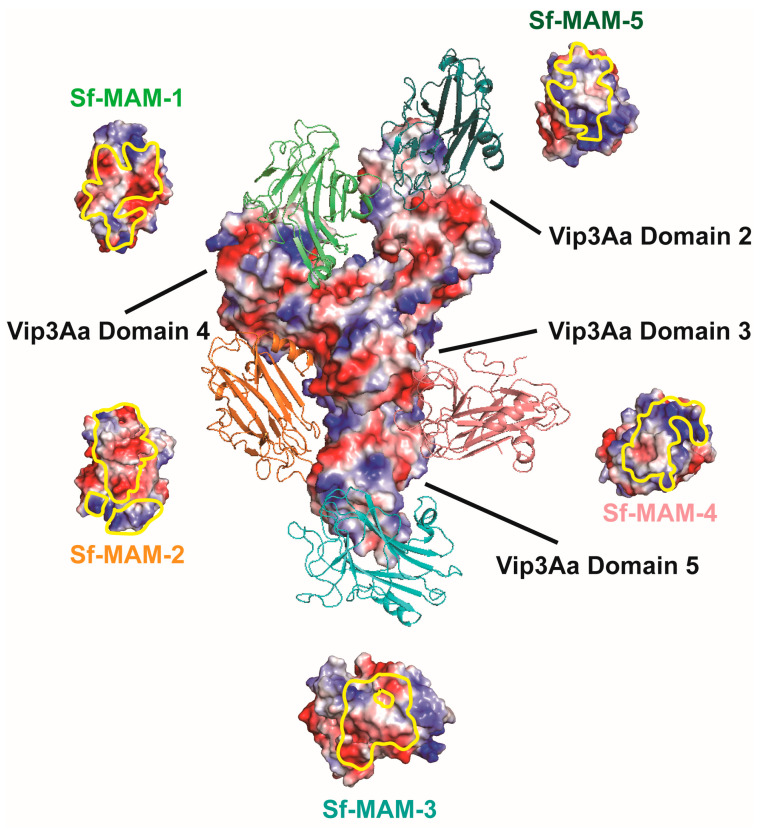
Structure docking models of Sf−MAM domain bound to Vip3Aa. The Sf-MAM domains in the five binding models are colored in green (Sf-MAM-1), orange (Sf-MAM-2), cyan (Sf-MAM-3), salmon (Sf-MAM-4) and teal (Sf-MAM-5), respectively. The PDB code for the Vip3Aa CTD structure is 6VLS. The domains of Vip3Aa are indicated by black lines. The yellow curved loop on Sf−MAM structure indicates the binding interface of Vip3Aa.

The full-length structure of Vip3Aa (Domains 1–5) is a tetramer with a long α-helix at the N-terminal, which maintains its stability (Figure S3a). The Vip3Aa CTD (Domains 2–5) was a Y-shaped structure with a putative receptor-binding domain (Figure S3b) [10,23]. After determining the structure of the Sf-MAM domain, we tried to dock the structure of the Sf-MAM domain to the structure of the Vip3Aa CTD (PDB ID: 6VLS) to acquire the putative binding models of the two proteins. We used the ZDOCK SERVER for protein–protein docking [22]. Briefly, two PDB files were both uploaded to the Website (https://zdock.umassmed.edu/, accessed on 15 June 2014), and ZDOCK (version 3.0.2) was selected to start the docking. The docking analysis generated five groups of models showing the binding of the Sf-MAM domain (1–5) to the Vip3Aa CTD (Figure 3). Among them, the contact surface electrostatic potential of Sf-MAM-2 (positive), Sf-MAM-3 (neutral) and Sf-MAM-5 (negative) was identical to the corresponding interface of the Vip3Aa CTD. Meanwhile, the contact surface electrostatic potential of Sf-MAM-1 (positive) and Sf-MAM-4 (negative) was complementary to the corresponding interface of the Vip3Aa CTD. As a former study had revealed that only Domain 2 and Domain 3 of Vip3Aa are involved in receptor binding [12], these five binding models of the Vip3Aa CTD bound to the Sf-MAM domain were all unreliable. The structure of the Sf-FGFR ectodomain has not been acquired in this study, and neither the entire Sf-FGFR nor its ectodomain structure have been reported yet. Therefore, Sf-FGFR/Vip3Aa docking was not conducted here.

### 3.4. In Vitro Binding of Vip3Aa to Sf-MAM and Sf-FGFR

**Figure 4:**
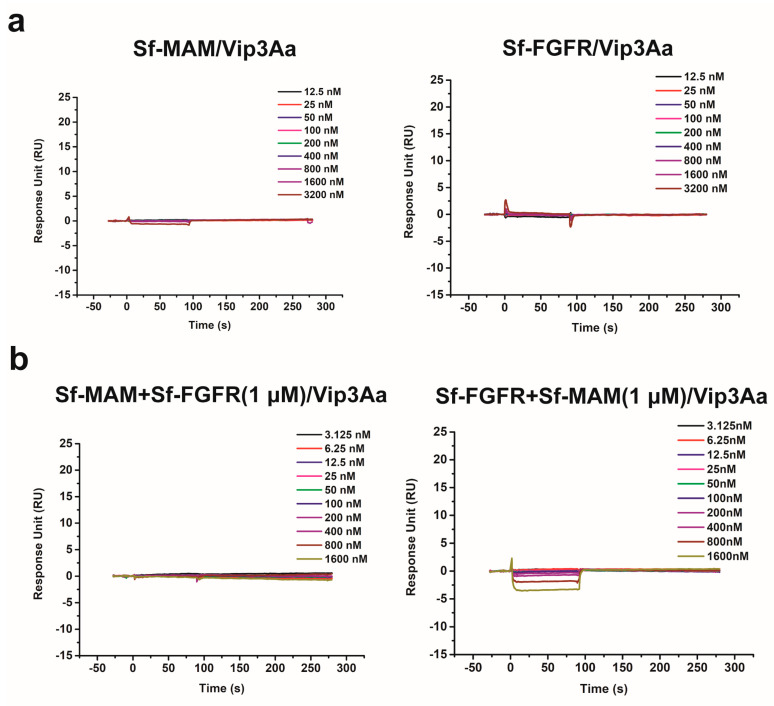
SPR binding curves of Vip3Aa to Sf−MAM, Sf−FGFR and Sf−MAM/Sf-FGFR mixture. (**a**) The binding curves of Vip3Aa bound to Sf-MAM domain (**left**) and Vip3Aa bound to Sf-FGFR ectodomain (**right**). (**b**) The binding curves of Vip3Aa bound to Sf-MAM/Sf-FGFR mixture. The raw curves are shown as colored lines. The concentration gradients of the Vip3Aa are displayed in the upper-right of the binding curves.

We then used the SPR assay to directly detect the binding affinity of Vip3Aa to Sf-MAM or the Sf-FGFR ectodomain. Briefly, the Sf-MAM domain or Sf-FGFR ectodomain was immobilized on a CM5 chip using sodium acetate solution (pH 5.0). Serial dilutions of Vip3Aa protein were flowed through. The curves showed that the binding signal could not be detected even if the concentration of Vip3Aa was as high as 3200 nM (Figure 4a). Previous studies detected the binding affinity of Vip3Aa to the Sf-MAM domain or Sf-FGFR ectodomain using an MST experiment [15,18]. But the Sf-MAM domain and Sf-FGFR ectodomain were both expressed in the *E. coli* system, which may have led to false-positive results.

In order to test the synergistic effect of the Sf-MAM domain and Sf-FGFR ectodomain on the interaction with the Vip3Aa protein, we immobilized the Sf-MAM domain on a CM5 chip while using 1 μM of Sf-FGFR and concentration gradients of Vip3Aa as the mobile phase, or immobilized the Sf-FGFR ectodomain on a CM5 chip while using 1 μM of Sf-MAM and concentration gradients of Vip3Aa as the mobile phase. The results showed that the coexistence of the Sf-MAM domain and Sf-FGFR ectodomain did not contribute to the interaction with the Vip3Aa protein (Figure 4b).

## 4. Discussion

As a second-generation insecticidal toxin secreted by *B. thuringiensis*, Vip3Aa has been widely used in commercial transgenic crops. Although the resistance allele frequency (RAF) for Vip3Aa is relatively low, the in-field monitoring of sentinel trials demonstrated that the RAF for Vip3Aa in *Helicoverpa zea* populations has been increasing [24,25]. The mechanism implicated in the binding of Vip3Aa to its cell receptor thus needs to be quickly revealed to better understand the insecticidal activity of Vip3Aa and improve the industrialization of Vip3Aa transgenic crops. In this study, we used the SPR binding assay to detect the interaction of Vip3Aa with its putative receptors, Sf-SR-C and Sf-FGFR. But no binding signal was detected between the Sf-MAM domain or Sf-FGFR ectodomain and Vip3Aa, which may indicate that the Sf-SR-C or Sf-FGFR protein could not serve as the cell receptor for Vip3Aa separately. But the possibility of Sf-FGER or Sf-SR-C having synergistic effects with other potential receptors on Vip3Aa entry could not be excluded. In addition, nine other proteins were found to interact with Vip3Aa by HPLC electrospray ion trap mass spectrometry (HPLC-MS/MS) [15,16]. Further studies are urgently needed to identify the genuine cell receptor of Vip3Aa and reveal the insecticidal mechanism of Vip3Aa.

The expression system may affect the properties of specific proteins. Sf-SR-C and Sf-FGFR are two membrane proteins on the Sf9 cell surface. The extracellular domains of these two proteins contain many glycosylation sites [17]. Expression of the two proteins in the *E. coli* system may cause misfolding due to a lack of post-translational modifications. In our study, we used Sf9 cells to express the Sf-MAM domain and Sf-FGFR ectodomain extracellularly, which may be closer to their natural state than proteins expressed by the *E. coli* system. This expression system difference may be an explanation for the controversial binding results.

In mammals, the main function of the scavenger receptor is endocytosis, which triggers a series of signal pathways linked to apoptosis [26,27]. But little research is available regarding the function of the SR-C protein described only in insects. As some pathogens can invade host cells by binding to scavenger receptors [28,29], our structure of the Sf-SR-C MAM domain may provide some insights into insect–pathogen interactions.

## Data Availability

The structural data have been deposited in the PDB website (https://www.rcsb.org) and are publicly available as of the date of publication. Accession number is 8YT7. This paper does not report original code. Any additional information required to reanalyze the data reported in this paper is available from the correspondence author J.L (lanjun2022@hnu.edu.cn) upon request.

## Supplementary Materials

The following supporting information can be downloaded at: https://www.mdpi.com/article/10.3390/insects15060428/s1, Table S1: Data collection and refinement statistics. Figure S1: The amino acid sequence of Sf-FGFR protein. Figure S2: Density map of Sf-MAM domain. Figure S3: Structure of Vip3Aa.
