# Supplementary material for: Binding Analysis of Sf-SR-C MAM Domain and Sf-FGFR Ectodomain to Vip3Aa
Source: Insects. 2024 Jun 6;15(6):428. doi: 10.3390/insects15060428 (PMC11203654; doi:10.3390/insects15060428)
Supplement: Supplementary file 1 [file insects-15-00428-s001.zip › insects-2965965-supplementary.pdf]

Supplemental information

Binding Analysis of Sf-SR-C MAM Domain and Sf-FGFR Ectodomain to Vip3Aa

Chenghai Wang [1,†], Min Li [2,†], Xiling Chen [2], Shilong Fan [2] and Jun Lan [1,*]

[1] School of biomedical sciences, Hunan University, Changsha 410082, China; wangch0529@hnu.edu.cn
[2] Beijing Advanced Innovation Center for Structural Biology, School of Life Sciences, Tsinghua University, Beijing 100084, China; helen2019@mail.tsinghua.edu.cn (M.L.); xlchen9057@126.com (X.C.); fanshilong@mail.tsinghua.edu.cn (S.F.)
* Correspondence: lanjun2022@hnu.edu.cn
† These authors contributed equally to this work.

Table S1. Data collection and refinement statistics.

	Sf–MAM
**Data collection**	
Space group	$P3_2 2 1$
Cell dimensions	
a, b, c (Å)	57.02, 57.02, 159.93
α, β, γ (°)	90, 90, 120
Resolution (Å)	50-2.25 (2.34-2.25)
Unique reflections	14876
Rmerge	0.10(1.88)
I/sI	38.67(1.16)
Completeness (%)	99.7(100)
Redundancy	9.9(10)
CC1/2	0.997(0.551)
**Refinement**	
Resolution (Å)	42.02-2.25
No. reflections	14872
R_{work}/R_{free}	26.47/26.97
No. atoms	1577
Protein	1542
Ligand	29
Solvent	6
B-factors	69.52
Protein	70.30
Ligand	30.73
Solvent	57.25
R.m.s deviations	
Bond length (Å)	0.012
Bond angles (°)	1.45
Ramachandran favored (%)	94.62
Ramachandran allowed (%)	5.38
Ramachandran outliers (%)	0.00

Data in bracket are for the highest resolution shell.

Sf-FGFR

MSLAAIAFWR	EMPIAVILLL	ATGCLAQTRE	IVLDKVYTRV	EVPLNERVRL	SCGMRPSINK	60
STVLWYKDGV	LVDSQRSKYV	QQKHWLRIKS	FDYTDVGVFV	CKFDNDDATK	LSLKLEVKPN	120
PHNPENGRDH	QTDITQAVQP	EALSQVVNKT	QEEGLSLPDS	KRYGKEDDDE	RAEKDHPGYG	180
HIDEKQDYFP	PRFKHPTKLY	YMDMKPAGNS	IRMRCAAEGN	PTPNITWYKN	RSTPITRMFF	240
QPSYGKWSMG	MEELTKADNG	NYTCVVCNIL	GCIEHTLALH	VQERLYAKPV	LTAGADNQTV	300
LVGETARFSC	QFLTDMHPSV	YWMYFTRDET	FENVTVNPAD	VTLQQSMVYY	DKNKVVTSEN	360
PEDKPEHLTI	YNVTKEDEGW	YVCIALNSLG	NTTAKGYLTV	LESPPVQEAP	DHGKHTLLIN	420
ILTVVLGAMF	FVAAIIVVMI	CKKLKREKEQ	KQLAIETARA	VIVTHWTKKV	TVEKPQMNGS	480
PTATGEALLM	PVVKIEKQKL	QQVQNTTSDS	MMMSEYELPM	DIDWEVPRDS	LCIGKVLGEG	540
EFGKVVKAEC	VGILKPGMQS	VVAVKMLKEG	HTDAEMMALV	SEMEMMKMIG	KHVNIINLLG	600
CCTQDGPLYV	IVEYAPNGNL	REFLRNHRPG	NRYESPTEDL	KEKKTLTQKD	LVSFSYQVAR	660
GMEYLASRRC	IHRDLAARNV	LVSDDCILKI	ADFGLAKDVH	SNDYYRKKTE	GRLPVRWMAP	720
ESLYHKVFTT	QTDVWSFGVL	LWEIMTLGGT	PYPTVPGQYM	YQHLSAGHRM	EKPPCCSLEI	780
YMLMRECWSF	SPGDRPSFTE	LVEDLDKILT	VTANQEYLDL	GLPQLDTPPS	SYDGSGDESD	840
SDFPFIK						847

Figure S1. The amino acid sequence of Sf–FGFR protein. The expression construct of Sf-FGFR ectodomain is marked in red.

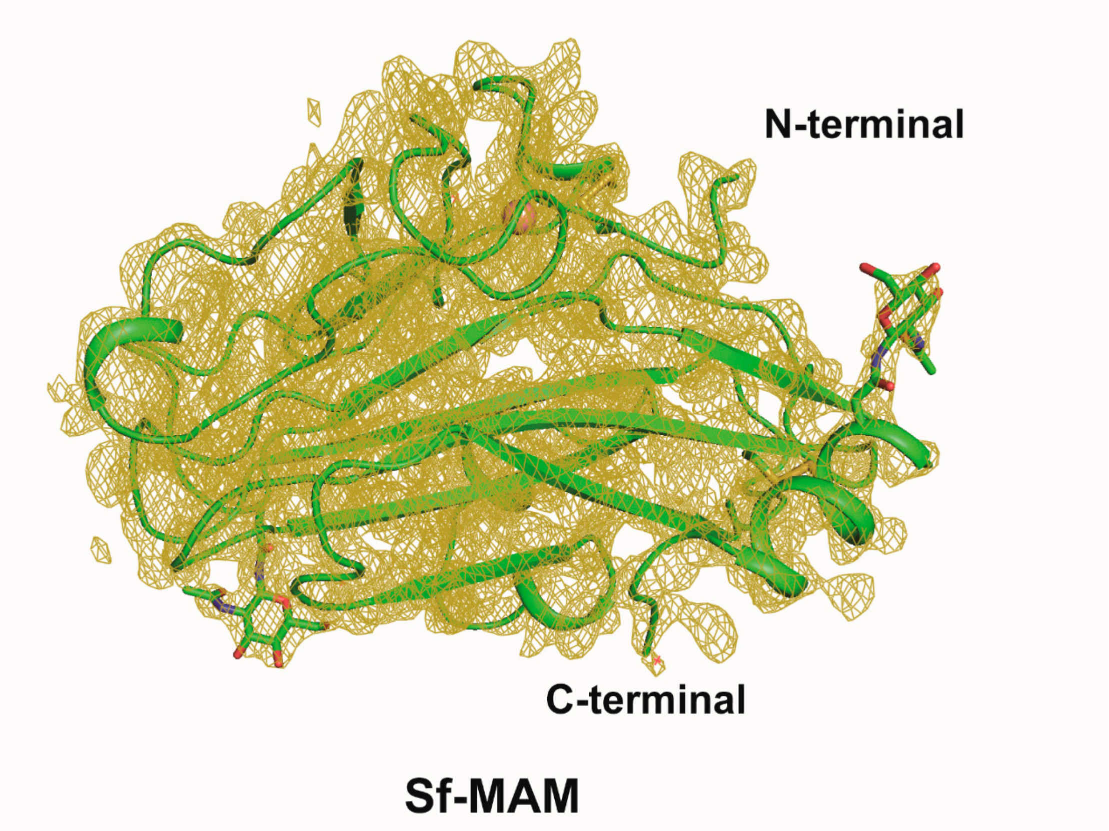

Figure S2. Density map of Sf–MAM domain. Sf-MAM is colored in green. Density map is colored in olive. Asparagine residues and glycan chains are shown as sticks. Calcium ion is shown as a salmon sphere.

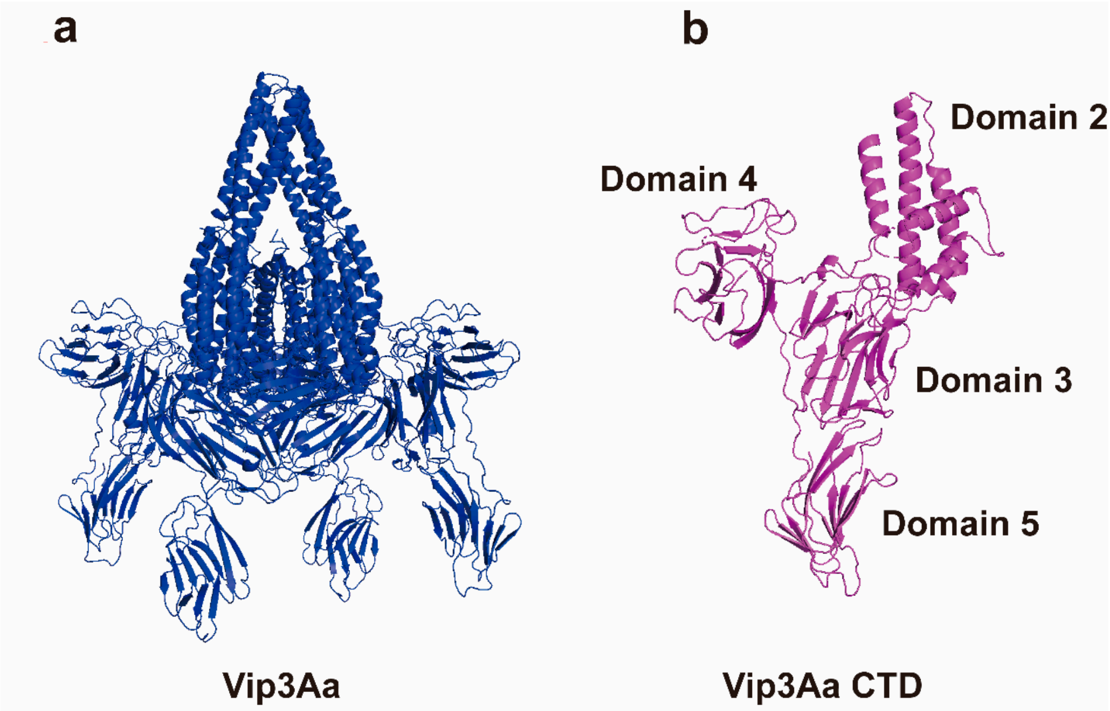

Figure S3. Structure of Vip3Aa. a, Structure of full-length Vip3Aa protein. The Vip3Aa structure is colored in blue. the PDB code for full-length Vip3Aa is 6TFJ. b, Structure of the Vip3Aa CTD. The Vip3Aa CTD structure is colored in magenta. the PDB code for Vip3Aa CTD is 6VLS.
